# Supplementary material for: Coding of interceptive saccades in parietal cortex of macaque monkeys
Source: Brain Struct Funct. 2021 Sep 1;226(8):2707–23. doi: 10.1007/s00429-021-02365-x (PMC8448682; doi:10.1007/s00429-021-02365-x)
Supplement: Supplementary file 5 — Supplementary file5 (DOC 626 KB) [file 429_2021_2365_MOESM5_ESM.docx]

Supplement


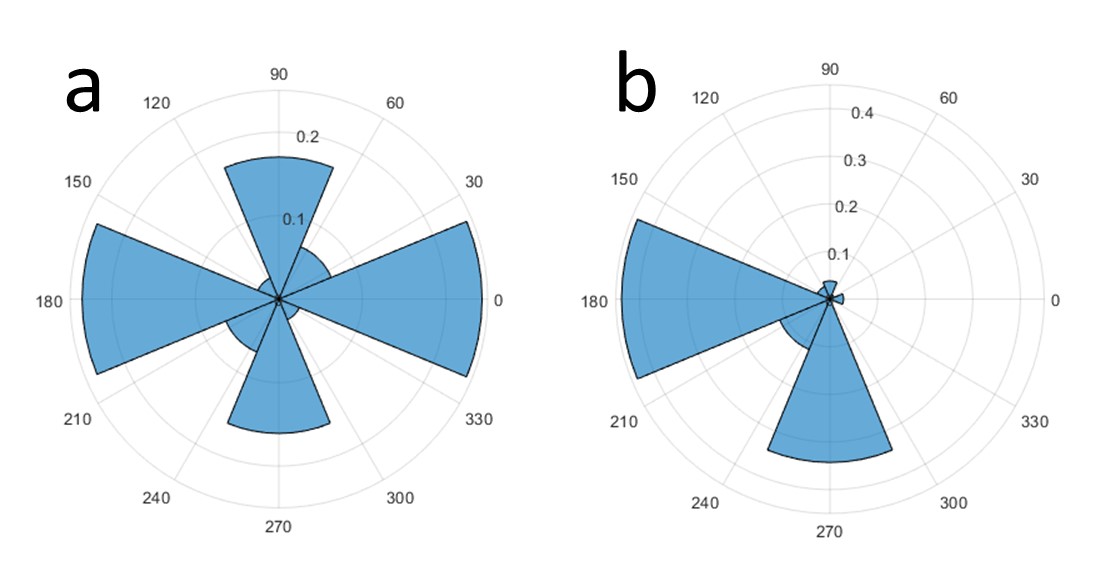


Figure S-1: Distribution of saccade directions (angles between the position of the fixation spot and the center of the screen) for all interceptive saccades including preferred and anti-preferred directions of each neuron (a) and including the preferred direction of each neuron only (b). Since the neural activities were recorded from the right hemisphere in both monkeys, the preferred saccade vectors were directed predominantly in the leftward (contraversive) direction. A substantial number of neurons also had a preferred vector in the downward direction.


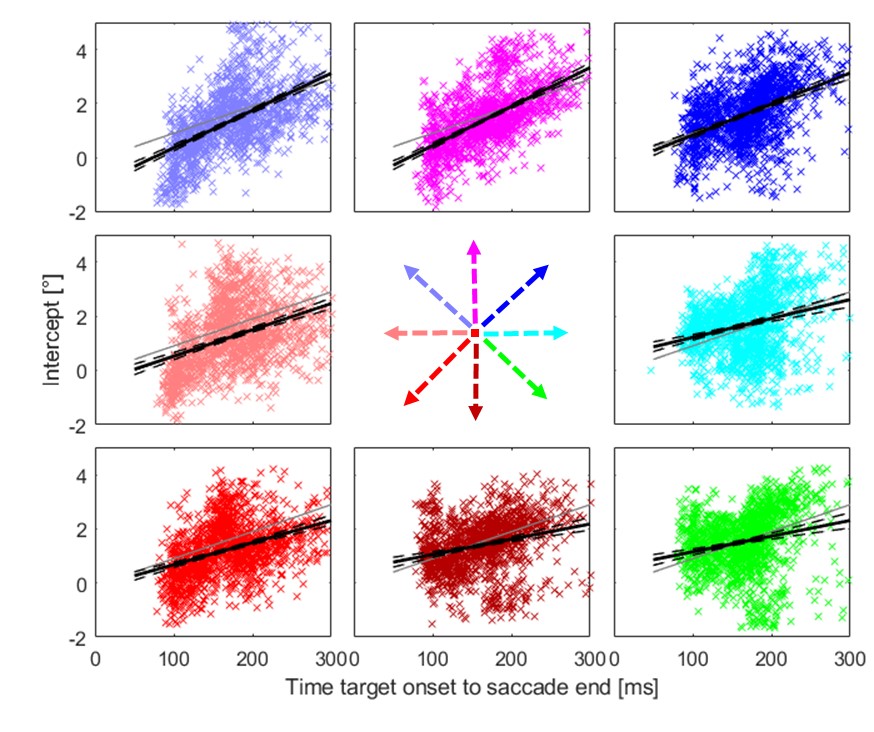


Figure S-2: Relationship between the timing of the end of the interceptive saccades and their intercepts for different directions of target motion. Different plots show the data from individual target directions according to the arrows. Grey lines marks the position of the target (relative to the center of the screen) at the respective time. Black line shows the results of a linear regression of the data and the black dashed lines its confidence interval (p=0.01, as calculated from a bootstrapping procedure).


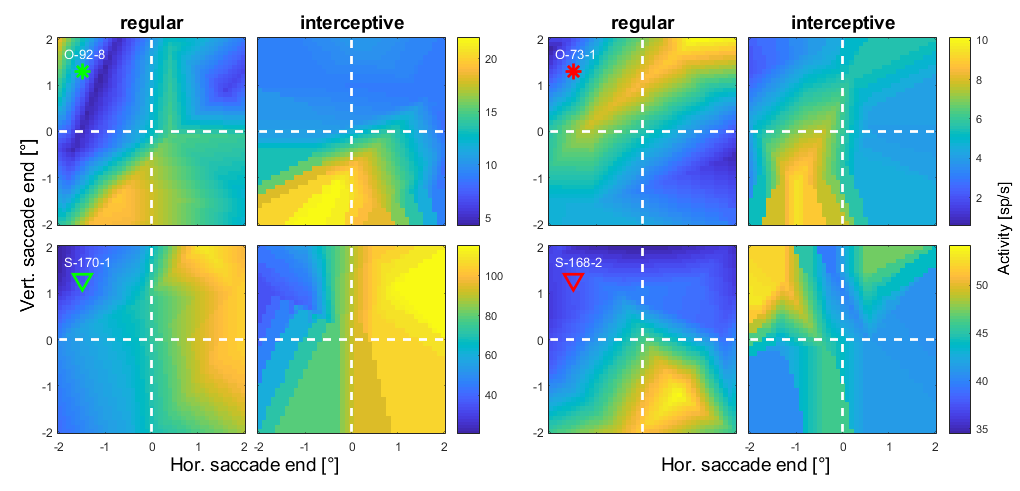


Figure S-3: Spatial tuning profiles from another four example neurons in the same format as Figure 6. The positions at the x and y axes represent the locations of saccade endpoints relative to the center of the screen. The two examples in the left two columns show an apparent similarity between the two activity profiles while for the neurons in the two columns on the right no such similarity was observed. Red and green markers below the cell-codes indicate the positions of each example neuron in the distribution of similarity indices shown in Figure 7.


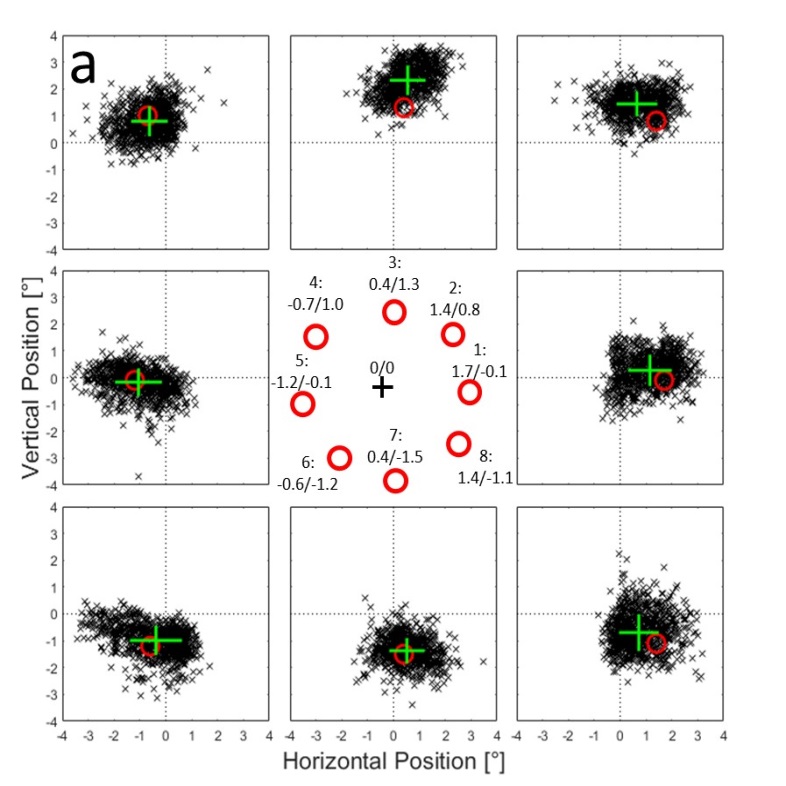

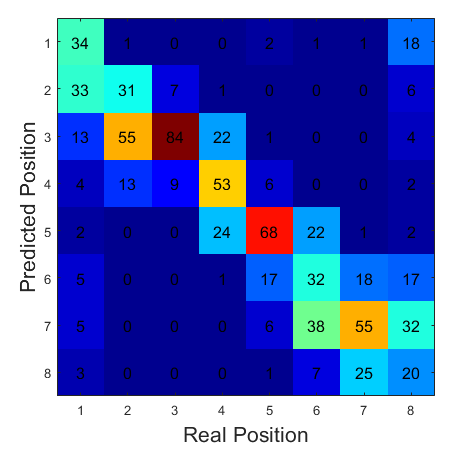


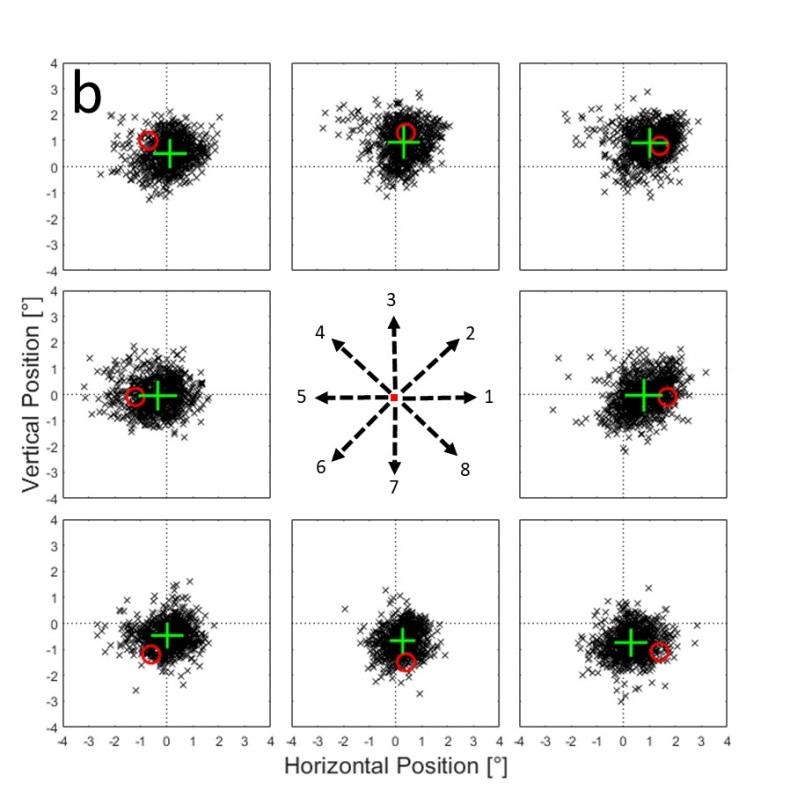

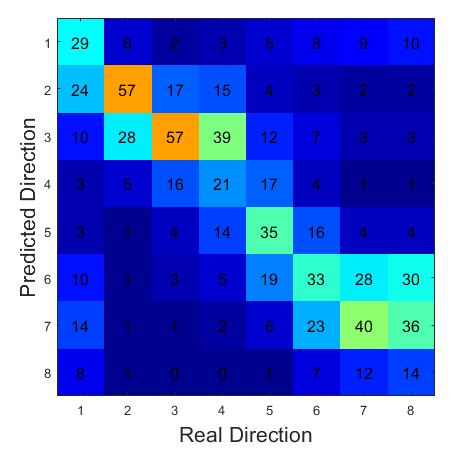


Figure S-4a: Example of the predictions from one neural network using estimated spike counts in a time window between 100 ms before and 100 ms after the onset of regular saccades. Black crosses represent the predicted saccade end points for 1000 single trials that were generated from data at positions marked by the red circles. Means and standard deviations in horizontal and vertical directions are shown as green crosses. In the confusion matrix on the right, the predictions from single trials over all validation samples were assigned to one of the tested positions based on the minimal Euclidian distance between the prediction and each of the tested positions. The numbers indicate the percentages of assignments for each tested 'real' position. b: Predictions of same network as in a) for saccade end-points of interceptive saccades.


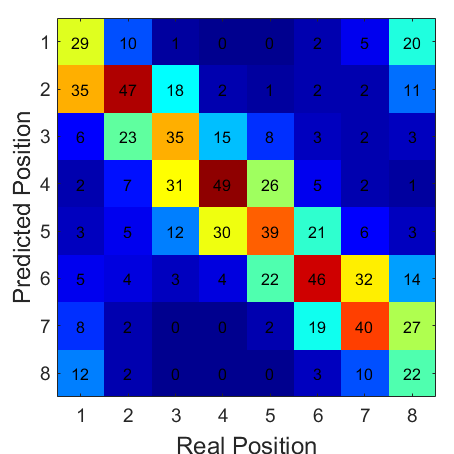

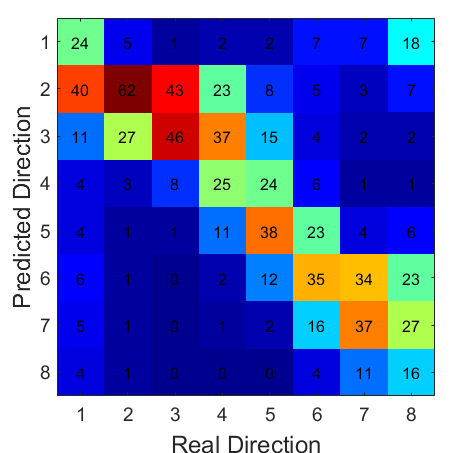


Figure S-5: Confusion matrices for results shown in Figure 10a, b. The saccade end positions were predicted based on the activity of a sample of 25 neurons that have previously shown the strongest similarity between the activity profiles for regular and interceptive saccades. They were then assigned to one of the eight tested positions based on minimal Euclidian distance. For regular saccades (left) on average 38% of the trials were assigned correctly and in 82% correct or to one of the neighbors. For interceptive saccades (right) on average 35% of the trials were assigned correctly and in 78% correct or to one of the neighbors.
